# Cell- and Tissue-Specific Transcriptome Analyses of *Medicago truncatula* Root Nodules

**DOI:** 10.1371/journal.pone.0064377

**Published:** 2013-05-29

**Authors:** Erik Limpens, Sjef Moling, Guido Hooiveld, Patrícia A. Pereira, Ton Bisseling, Jörg D. Becker, Helge Küster

**Affiliations:** 1 Laboratory of Molecular Biology, Wageningen University, Wageningen, The Netherlands; 2 Division of Human Nutrition, Wageningen University, Wageningen, The Netherlands; 3 Plant Genomics, Instituto Gulbenkian de Ciência, Oeiras, Portugal; 4 Institut für Pflanzengenetik, Leibniz Universität, Hannover, Germany; University of Nottingham, United Kingdom

## Abstract

Legumes have the unique ability to host nitrogen-fixing Rhizobium bacteria as symbiosomes inside root nodule cells. To get insight into this key process, which forms the heart of the endosymbiosis, we isolated specific cells/tissues at different stages of symbiosome formation from nodules of the model legume *Medicago truncatula* using laser-capture microdissection. Next, we determined their associated expression profiles using Affymetrix Medicago GeneChips. Cells were collected from the nodule infection zone divided into a distal (where symbiosome formation and division occur) and proximal region (where symbiosomes are mainly differentiating), as well as infected cells from the fixation zone containing mature nitrogen fixing symbiosomes. As non-infected cells/tissue we included nodule meristem cells and uninfected cells from the fixation zone. Here, we present a comprehensive gene expression map of an indeterminate Medicago nodule and selected genes that show specific enriched expression in the different cells or tissues. Validation of the obtained expression profiles, by comparison to published gene expression profiles and experimental verification, indicates that the data can be used as digital “*in situ*”. This digital “in situ” offers a genome-wide insight into genes specifically associated with subsequent stages of symbiosome and nodule cell development, and can serve to guide future functional studies.

## Introduction

Legume plants have the unique ability to host nitrogen-fixing bacteria, collectively called rhizobia, in a newly formed organ, the so-called root nodule. Inside specialized cells of the nodule, the rhizobium bacteria are accommodated as novel organelle-like structures called symbiosomes [1]. Symbiosomes fix atmospheric nitrogen into ammonium which is transferred to the plant in return for carbohydrates [2]. This symbiosis is one of the most important sources of biologically fixed nitrogen and allows legumes to grow in nitrogen poor soil conditions, without the need of chemical fertilizer. To better understand this ecologically and agriculturally important interaction a key goal is the identification of the transcriptome changes that are associated with the different stages of the interaction and to link gene expression to the corresponding developmental processes. One of the key processes that occurs in the nodule, and is at the heart of the symbiosis, is the accommodation and development of the bacteria into nitrogen-fixing symbiosomes. Here, we aim to characterize the transcriptome of specific cells/tissues inside the nodule at different stages of symbiosome formation in the model legume *Medicago truncatula* (Medicago). The developmentally structured organization of Medicago nodules makes them an ideal system to study the different stages of nodule and symbiosome development.

Nodule development is triggered by rhizobial lipochito-oligosaccharide signal molecules, called Nod factors that activate a signaling cascade which triggers transcriptional responses that control nodule organogenesis as well as rhizobial infection and symbiosome formation [3]. Rhizobia enter the root and developing nodule through tubular structures called infection threads. Typically, these infection threads originate in root hairs that curl around attached bacteria after which they traverse the cortex to deliver the bacteria to the developing primordium [4]. When the infection threads reach the cells of the nodule primordium, the bacteria are released from the cell wall bound infection threads and are taken up into the cells through an endocytosis-like process by which they become surrounded by a specialized plant membrane and organelle-like symbiosomes are formed [5]. After the infection threads invade the nodule primordium, an apical meristem is established that continues to add cells to the developing nodule [6]. In Medicago, this meristem stays active by which an elongated indeteminate nodule is formed. These nodules show a strictly organized zonation, where infection thread formation followed by symbiosome formation and subsequent development occur along a developmental gradient [7].

Zone I of the nodule consists of the apical nodule meristem, consisting of uninfected dividing cells. In Zone II, the infection zone, plant and bacterial cell differentiation occur and this zone can be further divided into a distal and proximal region [7]. In the distal infection zone, ∼4 cell layers just below the meristem, infection threads invade the cells coming from the meristem. Here so-called unwalled infection droplets extrude from the cell wall bound infection threads from where the bacteria are individually pinched off into the cytoplasm by which they become surrounded by the plant-derived symbiosome membrane [8], [9]. Next, the bacteria (now called bacteroids) divide and start filling the cells. In Medicago, bacteroid and symbiosome membrane division are strictly coupled by which symbiosomes remain single bacteria-containing compartments. In the proximal ∼4 cell layers of the infection zone, the bacterioids lose their ability to divide and start elongating. This terminal differentiation process has been correlated with endoreduplication and cell enlargement occurring in both the host cell as well as the bacteria and involves a family of nodule-specific cysteine-rich NCR peptides [10], [11]. In this way the individual symbiosomes become >10x bigger and almost completely fill the host cells. In Zone III, the fixation zone, the bacteria are fully differentiated into their nitrogen fixing form and nitrogen fixation takes place, which is facilitated by the micro-aerobic conditions in the infected nodules cells and correlates with the induction of bacterial nitrogen fixation genes [12], [13]. Some cells originating from the meristem never become infected by the bacteria and these can be clearly seen as relatively small uninfected cells in between the large infected cells. These uninfected cells are thought to play an essential role in metabolite transport to and from the infected cells [14]. Eventually, as the nodule ages (∼3–4 weeks post-inoculation), the symbiosis starts to break down and senescence of both symbiosomes and host cells occurs in Zone IV (senescent zone) [15]. The different zones mentioned above, except for the meristem, are surrounded at the periphery by the nodule parenchyma (nodule inner cortex), vascular bundles and the nodule endodermis. Further, the entire nodule is surrounded by an outer cortex [7].

In the past years, various expression profiling strategies have been used during both early and late stages of nodulation to identify the genes that are associated with different stages of the interaction [16]–[25]. Such studies either focused on identifying transcriptome changes within hours of treatment with symbiotic signals, with Rhizobium inoculation, or compared whole nodules at different time points after inoculation. To establish a link between gene expression and processes in the nodule, such as meristem formation, symbiosome formation, differentiation or maintenance, two recent studies combined transcriptome analyses of wild-type Medicago nodules with that of nodules impaired in their development due to bacterial and plant mutations [26], [27]. This revealed several expression profiles that correlated with distinct developmental programs in the nodule. However, this approach does not clearly distinguish between different cell types in the nodule. Furthermore, the use of plant and bacterial mutants has the inherent risk that genes are affected that are not normally expressed at corresponding developmental stages in wild-type nodules. Furthermore, genes that are differentially expressed in a specific cell type or at a specific stage might not be detected in whole nodule samples due to dilution effects by other more abundant cells.

Here, we used laser-capture microdissection (LCM) to isolate specific nodule cells at different stages of symbiosome development. To this end, we collected cells from the infection zone, divided into a distal region (where symbiosome formation and division occur) and a proximal region (where symbiosomes are mainly differentiating), as well as infected cells from the fixation zone containing mature nitrogen fixing symbiosomes. To include uninfected reference/control tissues, we also collected cells from the meristem as well as uninfected cells from the fixation zone. The captured cells/tissues were used to determine their associated expression profiles using Affymetrix Medicago GeneChips [22]. The resulting digital “*in situ*” offers a valuable data set to identify novel genes controlling nodule development and to unravel the unique ability of legumes to host the bacteria as nitrogen fixing organelles, which forms the heart of the Rhizobium-legume symbiosis, at a molecular level.

## Results and Discussion

### Laser Capture Microdissection of Medicago Nodules

**Figure 1:**
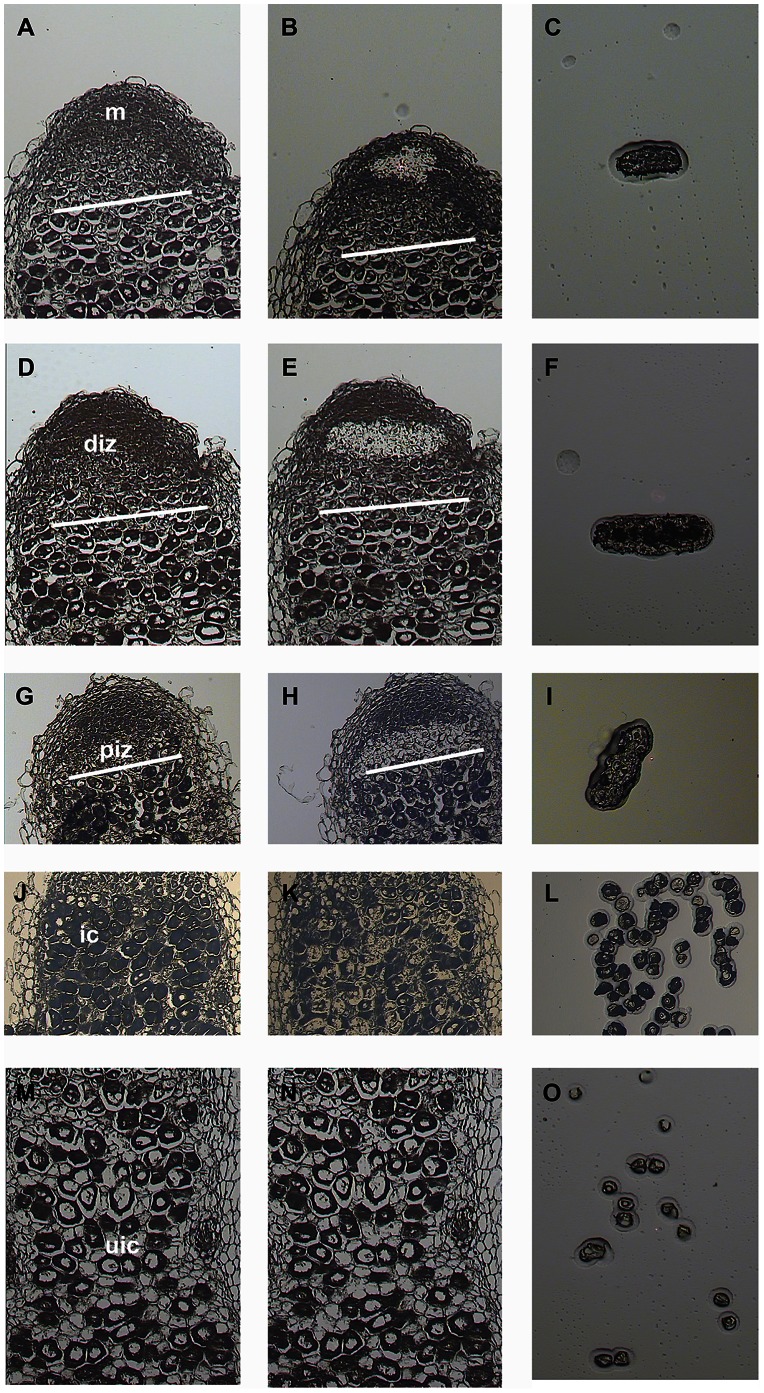
Laser capture microdissection of nodule cells. Panels represent 8 µM thick longitudinal sections of 3-week old Medicago nodules before capture (a,d,g,j,m), after capture (b,e,h,k,n) and captured/isolated cells (c,f,i,l,o). Cells/tissues were isolated from the meristem (m; a–c), distal infection zone (diz; d–f), proximal infection zone(piz; g–i), infected cells (ic; j–l) and uninfected cells (uic; m–o) from the fixation zone.

To isolate distinct nodule cells at different stages of symbiosome development we used LCM, which allows the rapid and specific isolation of cells/tissues based on conventional histological identification [28]. To preserve as best as possible the zonation and histological detail, three week old nodules were fixed with Farmer’s fixative and embedded in paraffin (according to [29]). Three week old Medicago nodules typically contain an active meristem at the apex, a well-defined infection zone and an active fixation zone. The quality of the RNA in the paraffin embedded tissues was checked before and after fixation and sectioning. Approximately 300 ng high quality total RNA could be isolated from a single paraffin embedded nodule (data not shown). Subsequently, 8 micrometer thick median longitudinal sections were used to isolate cells from the meristem, ∼4 cell layers of the distal infection zone (DIZ), ∼4 cell layers from the proximal infection zone (PIZ), infected (IC), and uninfected (UIC) cells from the fixation zone (Fig. 1a–o). Only those sections were used that showed a well-defined zonation and where histological preservation was sufficient to allow the identification of the different cell types. However, as the exact borders between the meristematic cells and the cells of the distal infection zone and between the distal and proximal infection zones are difficult to distinguish precisely by light microscopy, it is possible that some overlap exists between these laser-captured tissues. The same holds for the uninfected cells, which are relatively small, highly vacuolated and have irregular shapes in between the large infected cells. For each tissue/cell-type 3 biological replicates (e.g. different nodules) each consisting of ∼50 cells pooled from 8 consecutive sections were collected and used for RNA isolation.

### Cell-type Specific Expression Profiling of Medicago Root Nodules

To determine the transcriptome of the isolated cell/tissue types we used the Affymetrix Medicago GeneChips, which contains 50900 Medicago probe sets representing the majority of genes in this species [22]. The RNA isolated from the LCM cells was amplified using a two-step RNA amplification protocol to obtain sufficient material for hybridization experiments (see methods).

Analyses of the expression levels of control genes (i.e. GAPDH), divided into 3′- and 5′-regions, showed that there is a bias towards the 3′-end of transcripts (data not shown). This can be due to the two-step T7 amplification protocol and/or due to degradation of RNA in the LCM samples. Each gene on the Medicago GeneChip is represented by 11 probes. To account for this 3′-bias we re-analyzed the data using only the five most 3′ located probe sets instead of all 11 probe sets. Expression data are available at the Gene Expression Omnibus (GEO accession GSE43354). Although most probe sets on the Medicago array are designed in the 3′part of transcripts, the observed 3′-bias may affect the reliable detection of genes for which the probe sets on the array are located in more 5′ regions [30].

**Figure 2:**
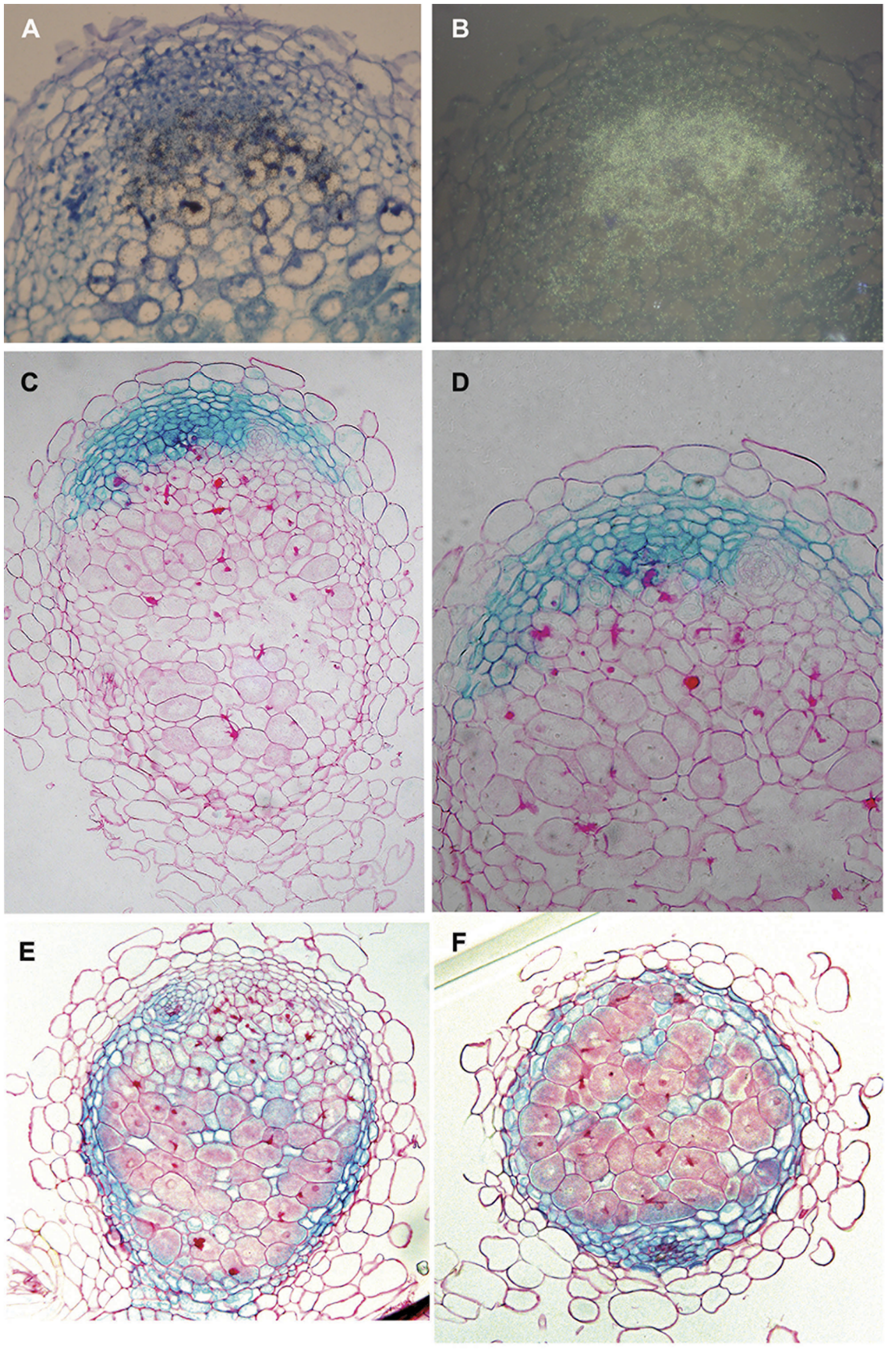
LCM data validation. (a,b) *In situ* localization of *MtENOD12* (antisense probe) in the infection zone of longitudinal sections of 14-day-old Medicago nodules, representing brightfield (a; signal appears as black dots) and epipolarization images (b). (c,d) Promoter-GUS analysis of Medicago ROP GTPase (Mtr.35940.1.S1_at, Mtr.15539.1.S1_at), showing β-glucoronidase (GUS) activity in the nodule meristem. (e,f) Promoter-GUS analysis of *MtENOD8.2*, showing β-glucoronidase activity in the non-infected cells of the nodule as well as in the nodule parenchyma.

To identify genes specifically correlated with the developmental processes in the different cell/tissue types we first selected genes that show enriched expression, at least 2-fold higher (q <0,1 (p<0.01)), compared to (the average of) all other LCM samples in: 1) the meristem (M), 2) the distal infection zone (DIZ), 3) the proximal infection zone (PIZ), 4) the complete infection zone (DIZ and PIZ), 5) infected cells (IC) of the fixation zone, and 6) uninfected cells (UIC). This data set is represented as Table S1. Figure 2 summarizes the number of genes that show at least 2 fold enriched expression compared to the average of the other LCM samples.

From this analysis, we next selected those genes that show at least a 2 fold higher enrichment factor in an individual/specific cell-type compared to any of the other cell types (Tables S2, S3, S4, S5, S6, S7). These genes will be referred to as “cell-type enriched” genes and are summarized in Table 1. In total 4999 genes show at least 2-fold enriched expression in a specific nodule tissue/cell type.

**Table 1:** Number of genes showing cell/tissue specific or enriched expression.

Meristem	Distal infection zone	Proximal infection zone	Total infection zone	Infected cells	Uninfected cells
**895**	**53**	**70**	**299**	**1909**	**2072**
**Total: 4999 genes**					

### Validation of Cell-type Specific Expression in Medicago Root Nodules

To validate the “specificity” of the obtained digital expression profiles and to establish to what extent the array data can be used as digital “*in situ*” we first compared the LCM microarray data to published expression profiles from promoter-reporter analyses or *in situ* hybridizations (summarized in Table 2). Additionally, we analyzed the expression profile of several selected genes in the nodule (Table 2).

**Table 2:** Selection of genes with known expression profiles used for validation.

Nodulecell/tissue-type	Gene	Reference	MedicagoGeneChip ID	AMean	Meristem	pvalue	qvalue	Distalinfectionzone	pvalue	qvalue	Proximalinfectionzone	pvalue	qvalue	Infectionzone	pvalue	qvalue	Infectedcell	pvalue	qvalue	Uninfectedcell	pvalue	qvalue
**Meristem**	MtWOX5	Dolgikh et al., 2011	Mtr.33304.1.S1_at	3.44	**42.87**	0.00	0.00	**1.28**	0.65	0.88	**0.47**	0.16	0.75	**0.71**	0.43	0.68	**0.21**	0.01	0.05	**0.19**	0.01	0.07
	MtHAP2	Combier et al., 2006	Mtr.43750.1.S1_at	12.32	**1.10**	0.74	0.69	**2.74**	0.00	0.28	**2.06**	0.02	0.53	**3.17**	0.00	0.02	**0.26**	0.00	0.00	**0.61**	0.10	0.31
	MtLYK3	Limpens et al., 2005	Mtr.142.1.S1_s_at	3.88	**23.41**	0.00	0.00	**9.52**	0.00	0.13	**0.34**	0.04	0.61	**2.21**	0.07	0.39	**0.11**	0.00	0.01	**0.12**	0.00	0.01
	MtROP2	this study (Fig. 2)	Mtr.35940.1.S1_at	2.23	**35.44**	0.00	0.01	**1.59**	0.54	0.86	**0.39**	0.22	0.78	**0.73**	0.61	0.75	**0.12**	0.01	0.05	**0.39**	0.22	0.43
**Infection zone**	MtN1	Gamas et al., 1998	Mtr.37500.1.S1_at	8.68	**0.40**	0.36	0.57	**20.66**	0.01	0.40	**25.73**	0.00	0.34	**65.62**	0.00	0.01	**0.05**	0.01	0.04	**0.10**	0.03	0.19
	MtN6	Mathis et al., 1999	Mtr.43850.1.S1_at	9.21	**6.10**	0.00	0.02	**29.22**	0.00	0.00	**17.25**	0.00	0.01	**63.33**	0.00	0.00	**0.02**	0.00	0.00	**0.02**	0.00	0.00
	DNF1	Wang et al., 2010	Mtr.43876.1.S1_at	12.35	**0.42**	0.00	0.00	**1.52**	0.01	0.42	**2.05**	0.00	0.06	**2.13**	0.00	0.00	**1.25**	0.13	0.25	**0.61**	0.00	0.04
	MtN9/MtMMPL1	Combier et al., 2007	Mtr.43552.1.S1_at	6.96	**0.36**	0.09	0.36	**6.40**	0.01	0.37	**15.64**	0.00	0.09	**21.55**	0.00	0.00	**2.59**	0.11	0.24	**0.01**	0.00	0.00
	MtEFD	Vernie et al., 2008	Mtr.41581.1.S1_at	9.14	**0.11**	0.00	0.02	**4.94**	0.01	0.39	**5.64**	0.00	0.33	**9.19**	0.00	0.01	**1.57**	0.38	0.44	**0.21**	0.01	0.08
	MtENOD12	this study (Fig. 2)	Mtr.8924.1.S1_at	7.82	**1.40**	0.62	0.66	**17.50**	0.00	0.19	**12.15**	0.00	0.28	**35.63**	0.00	0.00	**0.14**	0.01	0.06	**0.02**	0.00	0.00
**Distal**	MtENOD11	Journet et al., 2001	Mtr.13473.1.S1_at	7.95	**26.21**	0.00	0.02	**59.65**	0.00	0.04	**1.99**	0.34	0.81	**24.15**	0.00	0.01	**0.02**	0.00	0.00	**0.02**	0.00	0.00
**Infection zone**	MtERN1	Middleton et al., 2007	Mtr.7556.1.S1_at	7.08	**20.65**	0.00	0.00	**52.07**	0.00	0.00	**6.42**	0.00	0.17	**48.16**	0.00	0.00	**0.01**	0.00	0.00	**0.01**	0.00	0.00
	MtERN2	Middleton et al., 2007	Mtr.43947.1.S1_at	3.23	**1.15**	0.68	0.68	**5.09**	0.00	0.09	**0.68**	0.25	0.79	**2.28**	0.01	0.15	**0.35**	0.01	0.04	**0.71**	0.32	0.49
	MtAnn1	de Carvalho Niebel et al., 1998	Mtr.14183.1.S1_at	7.25	**0.15**	0.06	0.30	**4.51**	0.13	0.73	**1.35**	0.75	0.88	**3.34**	0.13	0.49	**1.65**	0.60	0.53	**0.66**	0.67	0.62
**Proximal**	MtIRE	Catalano and Dickstein., 2007a	Mtr.15644.1.S1_s_at	5.12	**0.13**	0.00	0.05	**3.34**	0.05	0.63	**7.22**	0.00	0.31	**8.35**	0.00	0.03	**2.27**	0.16	0.29	**0.15**	0.00	0.05
**Infection zone**	MtENOD20	Vernoud et al., 1999	Mtr.17106.1.S1_at	7.03	**0.08**	0.00	0.01	**11.26**	0.00	0.13	**26.86**	0.00	0.02	**45.06**	0.00	0.00	**0.74**	0.57	0.52	**0.06**	0.00	0.00
	MtGRPs	Kevei et al., 2002 (*M. sativa*)	Mtr.858.1.S1_s_at	3.72	**0.37**	0.03	0.23	**1.61**	0.28	0.80	**10.72**	0.00	0.05	**6.68**	0.00	0.01	**0.54**	0.17	0.29	**0.29**	0.01	0.10
	MtGRPs	Kevei et al., 2002 (*M. sativa*)	Mtr.49309.1.S1_at	4.05	**0.21**	0.03	0.21	**1.13**	0.85	0.90	**19.17**	0.00	0.11	**7.77**	0.00	0.06	**1.86**	0.35	0.42	**0.12**	0.00	0.06
**Infected cells**	Leghemoglobins	Ott et al., 2005	Mtr.38572.1.S1_at	9.23	**0.33**	0.00	0.02	**0.73**	0.25	0.79	**0.76**	0.30	0.81	**0.68**	0.08	0.43	**3.73**	0.00	0.00	**1.46**	0.16	0.38
	Leghemoglobins	Ott et al., 2005	Mtr.40138.1.S1_at	6.98	**0.25**	0.05	0.29	**0.42**	0.20	0.77	**0.62**	0.47	0.83	**0.41**	0.11	0.47	**5.93**	0.02	0.07	**2.59**	0.17	0.39
	Leghemoglobins	Ott et al., 2005	Mtr.43465.1.S1_at	9.76	**0.31**	0.00	0.03	**0.63**	0.12	0.73	**0.67**	0.17	0.75	**0.56**	0.02	0.26	**5.46**	0.00	0.00	**1.40**	0.25	0.45
	Leghemoglobins	Ott et al., 2005	Mtr.47990.1.S1_at	7.49	**0.16**	0.00	0.01	**1.05**	0.89	0.90	**0.48**	0.07	0.66	**0.63**	0.15	0.52	**5.71**	0.00	0.01	**2.19**	0.05	0.24
	Leghemoglobins	Ott et al., 2005	Mtr.5077.1.S1_at	2.73	**1.40**	0.22	0.49	**0.45**	0.01	0.42	**0.45**	0.01	0.42	**0.34**	0.00	0.02	**2.83**	0.00	0.01	**1.27**	0.38	0.51
	Leghemoglobins	Ott et al., 2005	Mtr.51231.1.S1_x_at	12.41	**0.35**	0.00	0.04	**0.85**	0.56	0.86	**0.78**	0.38	0.82	**0.76**	0.24	0.58	**3.54**	0.00	0.01	**1.23**	0.46	0.55
	Nodulin-26	Fortin et al., 1987	Mtr.2246.1.S1_at	8.05	**0.04**	0.00	0.05	**1.50**	0.64	0.88	**1.55**	0.62	0.86	**1.76**	0.43	0.69	**49.08**	0.00	0.01	**0.21**	0.09	0.30
	Nodulin-25	Hohnjec et al., 2008	Mtr.41813.1.S1_at	6.08	**0.17**	0.12	0.40	**0.36**	0.35	0.82	**0.64**	0.69	0.87	**0.38**	0.28	0.61	**43.45**	0.00	0.02	**0.58**	0.62	0.60
	MtNCR001	Van de Velde et al., 2010	Mtr.10380.1.S1_at	8.57	**0.55**	0.05	0.28	**0.63**	0.12	0.73	**0.58**	0.07	0.67	**0.51**	0.01	0.18	**3.96**	0.00	0.00	**1.26**	0.42	0.53
	MtNCR0035	Van de Velde et al., 2010	Mtr.10684.1.S1_at	12.07	**0.34**	0.00	0.06	**0.50**	0.04	0.60	**1.10**	0.75	0.88	**0.67**	0.13	0.49	**3.56**	0.00	0.01	**1.47**	0.22	0.43
	MtCaML2	Liu et al., 2006	Mtr.40731.1.S1_at	11.19	**0.38**	0.01	0.12	**0.58**	0.11	0.72	**0.56**	0.09	0.70	**0.47**	0.01	0.19	**5.23**	0.00	0.00	**1.58**	0.18	0.40
	MtCaML3	Liu et al., 2007	Mtr.37968.1.S1_at	7.36	**0.18**	0.02	0.19	**0.30**	0.09	0.69	**0.28**	0.07	0.67	**0.19**	0.01	0.15	**15.87**	0.00	0.01	**4.27**	0.05	0.23
	MtCaML6	Liu et al., 2008	Mtr.43719.1.S1_at	7.39	**0.09**	0.00	0.01	**0.41**	0.09	0.70	**0.38**	0.07	0.66	**0.29**	0.01	0.15	**30.96**	0.00	0.00	**2.34**	0.10	0.32
	MtIPD3	Messinese et al., 2007	Mtr.3453.1.S1_s_at	4.96	**0.20**	0.02	0.17	**0.40**	0.15	0.75	**0.28**	0.05	0.63	**0.23**	0.01	0.17	**19.26**	0.00	0.00	**2.32**	0.18	0.40
**Uninfected cells**	Asparagine synthetases	Shi et al., 1997 (*M. sativa*)	Mtr.8498.1.S1_at	11.95	**0.67**	0.62	0.66	**0.42**	0.29	0.80	**0.20**	0.06	0.65	**0.19**	0.02	0.26	**2.57**	0.25	0.36	**6.86**	0.03	0.18
	Asparagine synthetases	Shi et al., 1997 (*M. sativa*)	Mt.8499.1.S1_at	8.10	**0.34**	0.22	0.48	**0.27**	0.14	0.74	**0.18**	0.06	0.65	**0.13**	0.01	0.17	**1.75**	0.51	0.50	**33.86**	0.00	0.02
	Asparagine synthetases	Shi et al., 1997 (*M. sativa*)	Mtr.32211.1.S1_at	3.50	**0.25**	0.03	0.21	**0.44**	0.17	0.76	**0.26**	0.03	0.58	**0.24**	0.01	0.15	**3.57**	0.04	0.13	**9.77**	0.00	0.02
	Asparagine synthetases	Shi et al., 1997 (*M. sativa*)	Mtr.7084.1.S1_at	4.52	**0.57**	0.35	0.56	**0.67**	0.49	0.85	**0.56**	0.33	0.81	**0.52**	0.18	0.54	**0.73**	0.59	0.53	**6.44**	0.01	0.07
	MtbHLH1	Godiard et al., 2011	Mtr.10993.1.S1_at	7.33	**2.09**	0.22	0.49	**0.76**	0.64	0.87	**0.69**	0.54	0.85	**0.65**	0.38	0.66	**0.10**	0.00	0.01	**9.18**	0.00	0.03
	MtENOD8.2	this study (Fig. 2)	Mtr.8511.1.S1_at	4.39	**0.25**	0.02	0.16	**0.77**	0.61	0.87	**0.17**	0.00	0.33	**0.26**	0.01	0.13	**0.24**	0.01	0.07	**123.34**	0.00	0.00

Columns from left to right: Nodule cell/tissue-type, Gene name, Reference, Medicago GeneChip ID, Mean expression value (log2) in all tissues. For each cell/tissue: First column, Fold enrichment in that cell/tissue-type, Second column, corresponding p value, Third column, corresponding q-value statistics.

#### Meristem vs infection zone

First, we compared the meristem to the infection zone and surrounding cortex. The absence of *MtN13* (Mtr.33137.1.S1_at; Mtr.37852.1.S1_at) gene expression from the “meristem-enriched” data set, which is known to be highly expressed specifically in the nodule cortex [31], indicates that the meristem LCM sample is not significantly contaminated with nodule cortex cells (although some contamination can be observed in case of probe set Mtr.37852.1.S1_at). In addition, several genes that are reported to be specifically/most highly expressed in the infection zone were examined. These include for example *MtN1, MtN6, MtAnn1, DNF1/DAS12, MtRR4, MtN9/MtMMPL1* and *MtEFD* [31]–[37]. All these genes show infection zone enriched expression in the LCM samples (Table 2, Table S3), validating the results of the LCM analysis. As an additional example, we verified the infection zone-specific expression of the early nodulin *MtENOD12* (Mtr.8924.1.S1_at) by *in situ* hybridization. This showed that *MtENOD12* is indeed most highly expressed throughout the infection zone of the nodule and not (or hardly) in the nodule meristem (Fig. 2a,b).

To our knowledge, there are currently no genes described that are specifically/exclusively expressed in the nodule meristem of Medicago. At the switch from meristem to infection zone, the meristem-derived cells still enter the cell cycle, but instead of dividing they undergo several rounds of endoreduplication [38], [39]. Therefore, we looked whether genes associated with G2/M transition/cytokinesis are specifically enriched in the meristem data set. Indeed, the cytokinesis-specific t-SNARE/syntaxin *Knolle* (Mtr.41560.1.S1_at) and several cyclin and cyclin-dependent kinase genes that are required for G2/M transition (B-type cyclins: Mtr.31360.1.S1_at, Mtr.31859.1.S1_at; cyclin-dependent kinases (CDK2): Mtr.50839.1.S1_at, Mtr.43543.1.S1_at) show “meristem enriched” expression [39]–[41]. Among the genes that appear nodule “meristem enriched” is also the *WUSCHEL-RELATED HOMEOBOX5* gene (*MtWOX5*; Mtr.33304.1.S1_at), which is thought to also control stem cell activity in the root meristem. Recently, it has been shown by promoter- β-glucoronidase (GUS) reporter analyses that *MtWOX5* is indeed expressed in the nodule meristematic region, most specifically at the tips of the vascular bundles [42]. These cells may be related to meristem-organizing quiescent center cells, although the exact organization of the nodule meristem and stem cell niche is not known.

The Nod factor receptor LYK3 (Mtr.142.1.S1_s_at) also shows meristem enriched expression in the LCM data. Previous *in situ* hybridizations have shown that *LYK3* is expressed in the proximal site of the nodule meristem at the border with the infection zone, where it may control the invasion of the meristematic cells by infection threads [43]. To further confirm the predictive value of the “meristem enriched” data set, the putative promoter region of a ROP GTPase, (Mtr.35940.1.S1_at, Mtr.15539.1.S1_at), was isolated and its expression determined by promoter-GUS analysis. This confirmed the “meristem”-specific expression of this gene in Medicago nodules (Fig. 2c,d). These data indicate that the meristematic region as captured can be clearly distinguished from the infection zone.

#### Distal vs proximal infection zone

The infection zone can be further divided into a distal and proximal zone based on the developmental status of the symbiosomes in this part of the nodule. In the distal part (∼4 cell layers just below the meristem), after infection threads have invaded the meristem-derived cells, symbiosomes are formed (bacteria are released from the infection threads) and symbiosomes divide. In the proximal ∼4 cell layers symbiosomes have stopped dividing and are terminally differentiating by which they become much bigger and fill the growing nodule cells. To identify genes potentially associated with these different stages, we selected infection zone enriched genes that are >2x enriched in the distal infection zone compared to the proximal infection zone or vice-versa (Table S4, S5). Two genes have been shown to be most highly expressed in the distal infection zone. These are the early nodulin *ENOD11* (Mtr.13473.1.S1_at) and annexin *MtANN1* (Mtr.14183.1.S1_at) [32], [34]. Both genes show distal infection zone enriched expression in the LCM array data, confirming the specificity of the captured cells. Among the genes that show a “proximal infection zone enriched” expression (Table S5) is the nodule-specific *IRE* gene (Mtr.15644.1.S1_s_at). This AGC-like kinase has been shown to be most highly expressed in the proximal part of the nodule infection zone via promoter-GUS analyses [44]. Additional genes that have been reported to be expressed most highly in the proximal infection zone and which show enrichment in the proximal infection zone LCM data, include the phytocyanin-like *ENOD20* (Mtr.17106.1.S1_at) [45] and glycine-rich protein-encoding genes (*GRP*s; Mtr.858.1.S1_s_at, Mtr.49309.1.S1_at) [46]. These data validate the specificity of the LCM data to distinguish distal from proximal cells in the infection zone of the nodule.

#### Infected vs uninfected cells

The fixation zone consists of two cell types; infected and uninfected cells. To validate the specificity of the infected versus uninfected LCM data we looked for genes that are reported to be specifically expressed in either cell type in Medicago. Recently, a basic helix–loop–helix transcription, MtbHLH1, was shown to be expressed in Medicago nodules in the uninfected cells and vascular bundles, where it is thought to control vascular patterning and nutrient exchange [47]. In our LCM data *MtbHLH1* (Mtr.10993.1.S1_at) indeed shows specific expression in the uninfected cells, confirming the reported promoter-GUS data. To further validate the uninfected cell LCM data we checked the expression pattern of *MtENOD8.2* (Mtr.8511.1.S1_at), which shows anuninfected cell “specific” expression from the LCM data. ENOD8.2, like its close homolog ENOD8.1, belongs to the GDSL family of lipase and esterase proteins [48]. The putative promoter-region of *MtENOD8.2* was fused to β-glucoronidase and its expression pattern analyzed in nodules. This analysis confirmed the uninfected cell “specific expression” of *MtENOD8.2* (Fig. 2e,f). Additionally, *ENOD8.2* was found to be expressed in the nodule parenchyma. Therefore, the uninfected cell enriched data set presented here offers an important insight into this essential nodule cell type (Table S7).

Several genes have been reported that show specific/highly enriched expression in the infected cells of the fixation zone. These include: *Leghemoglobin* genes [13], aquaporin *Nodulin-26* [49], *Nodulin-25* [50], sulfate transporter *SST1* [51], NCRs including for example *NCR001/NCR035* [11], [52], and *Calmodulin-like/CaML* genes [53]. All these genes indeed show enriched expression in the LCM infected cells from the fixation zone (Table 2, Table S6) confirming the specificity of the LCM data.

### Cell/Tissue-specific Characteristics of Gene Expression in Medicago Root Nodules

Next, we examined the nodule cell/tissue-specific transcriptomes for characteristics that may be linked to the specific processes that occur in these cell types, with a special focus on symbiosome development and function.

#### “Infection zone enriched”

The infection zone data set may contain numerous candidate genes that control the formation and development of symbiosomes. The nodule-specific signal peptidase subunit MtDNF1/DAS12, the putative metallo-peptidase MtMMPL1 and the AP2/ERF transcription factor MtEFD have indeed been shown to control infection and symbiosome development in this part of the nodule. MtEFD has been shown to be able to induce the expression of the A-type cytokinin response regulator *MtRR4*, which is thought to negatively regulate cytokinin signaling [36]. *MtRR4* (Mtr.9656.1.S1_at) indeed shows specific expression in the infection zone, with highest expression in the proximal part (see transcriptional regulators below). Therefore, downregulation of cytokinin signaling in the infection zone may be required for proper differentiation of symbiosome and nodule cells.

Terminal symbiosome differentiation is triggered by nodule-specific cysteine-rich peptides (NCRs) that resemble antimicrobial peptides. These NCRs contain a N-terminal signal peptide, which is processed by a nodule specific signal peptidase complex containing DNF1 that is active in the infection zone of the nodule, by which these peptides are targeted to the symbiosomes via a secretory pathway [11], [37]. Most NCR peptides are specifically induced in the infected cells of the infection zone, as also determined by *in situ* hybridization or promoter-GUS analysis [11], [52]. However, several NCR encoding genes (Mtr.35829.1.S1_at, Mtr.29559.1.S1_at, Mtr.37119.1.S1_at) are specifically enriched in the infection zone, of which most tend to be higher expressed in the proximal part of the infection zone where terminal differentiation is observed (see also “proximal enriched” below). These NCRs may be key candidates that initiate the bacterial differentiation process.

#### “Distal infection zone enriched”

To identify “distal infection zone enriched” genes we selected infection zone enriched genes that are >2x enriched in the distal infection zone compared to the proximal infection zone (Table S4). Among these genes are *ENOD11* (Mtr.13473.1.S1_at), *ERN1* (Mtr.7556.1.S1_at), *ERN2* (Mtr.43947.1.S1_at) and *MtN2* (Mtr.3197.1.S1_at) [16], [34], [53], [54]. It has been shown that the AP2/ERF transcription factors ERN1 and ERN2 function in the Nod factor signaling pathway and bind to a conserved motif GCAGGCC (NF-box) in the promoter region of *ENOD11* where they act as transcriptional activators [53]. MtERN1 has been shown to be required for infection thread initiation and maintenance of infection thread growth in the epidermis [55]. Therefore, it can be hypothesized that ERN1 similarly controls infection events in the distal infection zone of the nodule through the activation of specific genes. It is known that the rhizobial *nod* genes involved in Nod factor production are still expressed by rhizobia inside infection threads in the distal infection zone of the nodule [56], [57], where also the Nod factor receptors are expressed [43] and that they are switched off as soon as the bacteria are released into the cells [58]. This suggests that Nod factor (NF) perception and signaling occur in these cells. To investigate whether this is also reflected in the LCM expression data, we compared the induction of genes 24 hours after NF treatment in the root (reported by [25]) with the genes specifically enriched in the apical part of nodule. This showed that ∼20% (10 genes) of the “distal infection zone specific” genes are also induced 24 hours after NF treatment in plantlet roots (Table S8), whereas only 1,5% (13) of the meristem specific genes or 4% (3) proximal infection zone specific genes are induced by Nod factor treatment there. This supports the hypothesis that NF signaling occurs at the transition from the meristem to the distal infection zone. However, overall, <10% of the 283 NF-induced genes show specific expression in the apical part of the nodule, which suggests that many of the 24 h NF-induced genes are specifically induced in root tissues.

An interesting gene that shows “specific” expression in the distal infection zone is the Medicago ortholog (Mtr.26489.1.S1_at) of a recently identified pectate lyase (LjNPL) in *Lotus japonicus*. LjNPL was shown to control infection thread formation revealing that the plant actively contributes to plant cell wall degradation to facilitate rhizobial infection [59]. Therefore, MtNPL may also be involved in infection thread formation in the nodule and/or the formation of unwalled infection droplets to allow symbiosome formation. The putative *MtNPL* promoter region does not contain a conserved NF-box, indicating that different/additional transcription factors control the induction of this gene, such as the putative transcription factor NIN which was shown to bind to the *LjNPL* promoter [59].

Another component that is implicated in rhizobial infection is the ARP2/3 complex which controls actin polymerization. Mutations in the SCAR/WAVE complex, involved in the activation of the ARP2/3 complex, block infection by rhizobia [60]. Among the distal infection zone enriched genes is a subunit of the ARP2/3 complex (Mtr.37170.1.S1_at). Interestingly, an ortholog of this subunit was recently shown in Lotus to control rhizobial infection [61]. Therefore, control of the actin cytoskeleton likely also plays a key role in the nodule to control infection thread formation and possibly symbiosome formation [62].

One of the most specifically expressed genes in the distal infection zone encodes a putative protease inhibitor, Mtr.35511.1.S1_at. This gene is also highly induced in arbuscular mycorrhizal (AM) roots, specifically in cells containing arbuscules [63]. It has recently become clear that rhizobia recruited the signaling pathway, including lipo-chitooligosaccharide signal molecules and receptor, from the ancient AM symbiosis to establish an intracellular symbiotic interface [64], [65]. Therefore, it is tempting to speculate that this protease inhibitor is involved in the intracellular accommodation of both symbionts. However, despite the shared signaling pathway there is overall only a limited overlap in genes that show enriched expression in mycorrhized roots and symbiosome containing nodule cells (Table S13).

#### “Proximal infection zone enriched”

Among the genes that show a “proximal infection zone enriched” expression (Table S5) is the nodule-specific AGC kinase gene *IRE* (Mtr.15644.1.S1_s_at) [44]. AGC kinases are key regulators of cell growth and MtIRE could potentially play an important role in symbiosome and/or nodule cell enlargement, possibly through the regulation of vesicle trafficking or cytoskeletal organization [66]. A list of (receptor-like) kinases enriched in the different cells or tissues is represented in Table S9.

Another striking (distal and) proximal infection zone specific gene is a close homolog of the CLAVATA1-related AtBAM3 receptor-like kinase (Mtr.4752.1.S1_at). In Arabidopsis BAM kinases regulate meristem function at shoot and flower meristems through complex interactions with CLAVATA signaling [67]. CLE peptides have been identified as ligands for such receptor-like kinases and another CLAVATA1 homolog, in Medicago called SUNN, has been shown to control nodule number in the process of autoregulation of nodule numbers [68]. It is therefore tempting to speculate that MtBAM3 plays a role in the perception of CLE peptides (such as the recently identified MtCLE12 and MtCLE13) in the nodule to control the balance between cell proliferation and differentiation.

As mentioned above, terminal symbiosome differentiation is triggered by nodule-specific NCR peptides [11]. Several NCR peptides appear most highly induced in the proximal part of the infection zone coinciding with the induction of symbiosome differentiation. These include: Mtr.4538.1.S1_at, Mtr.48527.1.S1_at and Mtr.10836.1.S1_at. Most of these NCR genes, including the infection zone-enriched NCRs Mtr.35829.1.S1_at, Mtr.29559.1.S1_at, Mtr.37119.1.S1_at, already show enriched expression in the distal infection zone. Therefore, these NCR’s may be key NCR peptides to initiate symbiosome differentiation.

Several genes involved in cytokinin signaling show highest expression in the proximal infection zone. These include a histidine phosphotransfer encoding gene (Mtr.11120.1.S1_at), two cytokinin-specific phosphoribohydrolase *LOG*s (Mtr.39530.1.S1_at, Mtr.50458.1.S1_at) which activate cytokinins [69], as well as two A-type RR genes (Mtr.9656.1.S1_at (*MtRR4*), Mtr.17273.1.S1_s_at) and a cytokinin oxidase (Mtr.14413.1.S1_at) that negatively regulate cytokinin signaling. Therefore, we speculate that cytokinin signaling is tightly regulated in the (proximal) infection zone of the nodule to control the proper differentiation of nodule cells and symbiosomes.

#### “Infected cell enriched”

Among the genes that show enrichment in the infected cells of the fixation zone is the essential Nod factor signaling gene *DMI1* (Mtr.19417.1.S1_at, Mtr.124.1.S1_s_at), which encodes a putative cation channel that is required to induce calcium-spiking upon Nod factor perception in the epidermis [70], [71]. DMI1 was also identified by Moreau and co-workers [27] as a late expressed gene in their transcriptome analyses. Furthermore, the interacting protein of DMI3, *IPD3* (Mtr.3453.1.S1_s_at) is most highly expressed in the infected cells of the fixation zone, as confirmed by promoter-reporter analyses [72], [73]. Also DMI3 was shown to be expressed throughout the infection zone up to the fixation zone [74]. This indicates that several components of the Nod factor signaling pathway are also active at relatively late stages in the nodules, most likely to control symbiosome development [73]. As the bacterial *nod* genes are not active in these cells it suggests that additional mechanisms, independent of Nod factor perception, are able to activate DMI3 in these cells.

A functional nitrogen-fixing symbiosis requires the efficient transport of metabolites to, and from, the nitrogen-fixing symbiosomes. The bacteroids require carbohydrates from the plant, which are mainly supplied in the form of dicarboxylic acids, especially malate [14]. In pea it has been shown that bacteroids also need to be supplied by branched-chain amino acids [75]. How these components are transported across the symbiosome membrane, resembling transport to the apoplast [9], is currently not known. Additional minerals that need to be supplied by the host cells are for example zinc, iron, magnesium and sulfate. Most of these components will need specific transporters on the symbiosome membrane to be transported to the bacteroids, as exemplified by the sulfate transporter *SST1* (Mtr.37708.1.S1_at) [51]. However, in most cases the transporters involved are not known. Therefore, we searched for putative transporters that are specifically enriched in the infected cells containing nitrogen-fixing symbiosomes (based on Mapman and GO classification). These “infected cell enriched” transporters are summarized in Table S10. Among the genes are putative candidates for the transport of malate (Mtr.13956.1.S1_at), zinc (Mtr.41323.1.S1_at, Mtr.32958.1.S1_at), nitrate (Mtr.40270.1.S1_at), potassium (Mtr.9837.1.S1_at) and several aquaporin-like proteins potentially transporting ammonium [76].

#### “Meristem enriched”

The nodule meristem was captured to serve as one of the uninfected reference/control tissues for the infected nodule cell types. However, in addition, the meristem enriched transcriptome gives first insight into molecular players that controls its organization.

Among the genes that appear nodule “meristem enriched” (Table S2) are many genes that are associated with meristematic/dividing cells. These include the *WUSCHEL-RELATED HOMEOBOX5* gene (*MtWOX5*; Mtr.33304.1.S1_at), *SCARECROW* (*MtSCR*; Mtr.39371.1.S1_at) and *BABY BOOM* gene (*MtBBM*; Mtr.21627.1.S1_at) which are known to control stem cell activity in the root meristem [77], [78]. This supports the hypothesis that nodule formation recruits a program involved in lateral root formation [79], [80]. Furthermore, the array data indicate an important role for auxin signaling in the control and maintenance of a functional nodule meristem. Several auxin signaling related genes show a “meristem specific” expression in the nodule. These include for example: **AUX/IAA’s** (Mtr.43054.1.S1_at, Mtr.38407.1.S1_at, Mtr.43345.1.S1_at, Mtr.10432.1.S1_at, Mtr.48811.1.S1_at, Mtr.13714.1.S1_at, Mtr.41219.1.S1_at, Mtr.33279.1.S1_at), **ARF’s** (Mtr.26217.1.S1_at, Mtr.35827.1.S1_at, Mtr.11167.1.S1_at, Mtr.39377.1.S1_at, Mtr.24462.1.S1_at, Mtr.44217.1.S1_at), **TIR1**-like F-box (Mtr.37555.1.S1_at), **PIN** auxin efflux carriers (Mtr.45124.1.S1_at, Mtr.38716.1.S1_at) and auxin responsive genes such as **GH3**-like (Mtr.6663.1.S1_at, Mtr.40094.1.S1_at, Mtr.41237.1.S1_at) and **SAUR**-like genes (Mtr.20120.1.S1_at, Mtr.19927.1.S1_x_at). The importance of auxin in the nodule meristem was also suggested from the activation of auxin responsive promoters in the nodule meristem [81]. Furthermore, auxin signaling has been linked to the control of nodule numbers in the process of autoregulation. One of the genes that is highly expressed in the nodule meristem is the Medicago ortholog (Mtr.43054.1.S1_at) of *IAA14/SLR* (*SOLITAIRY ROOT*), which has been shown to control lateral root formation [82], [83]. In Arabidopsis, a stabilizing mutation in IAA14 blocks lateral root formation by inhibiting the auxin response factors ARF7 and ARF19 [82], [84]. Interestingly, several mutants, such as the pea *cochleata* and Medicago *noot* mutant, have been identified where the nodule meristem switches to a root meristem and roots emerge from the nodules [79], [80]. Therefore, the upregulation of *IAA14* expression in the nodule meristem may play a role in inhibiting the switch to a lateral root meristem. However, given the number of auxin-related signaling genes, auxin signaling in the nodule meristem is likely to be a complex process involving various feedback loops.

#### “Uninfected cell enriched”

As an additional reference cell-type uninfected cells from the fixation zone were captured. Although uninfected cells are thought to play an essential role in metabolite transport in functional nodules. The uninfected cell enriched data set presented here offers a first insight into this essential nodule cell type (Table S7).

Carbon derived from photosynthesis is transported mainly as sucrose via the phloem, which is thought to be cleaved/converted in the uninfected nodule cells into malate to be transferred to the infected cells [14]. In support of this, several genes involved in sucrose cleavage and transport are found specifically/enriched in the uninfected cells. These include: putative *SWEET/MtN3*-like sucrose transporters (Mtr.42041.1.S1_at, Mtr.43349.1.S1_at, Mtr.8585.1.S1_at, Mtr.41025.1.S1_at) [85], sucrose/H+ co-transporters (Mtr.21349.1.S1_s_at, Mtr.12339.1.S1_at), sucrose synthases (Mtr.2239.1.S1_at, Mtr.43674.1.S1_at, Mtr.22018.1.S1_s_at), and a sucrose-cleavage protein (Mtr.43417.1.S1_at).

Uninfected cells in legumes such as Medicago are further thought to play a role in the transport/export of fixed nitrogen in the form of amides, of which asparagine is considered to be the major exported nitrogenous compound [14]. Several genes involved in asparagine synthesis, such as *ASPARAGINE SYNTHETASE*, have been found to be highly expressed in uninfected cells in alfalfa, in addition to their expression in infected cells [86]. The LCM array data show that also in Medicago asparagine synthase (Mtr.8498.1.S1_at; Mtr.8499.1.S1_at; Mtr.32211.1.S1_at; Mtr.7084.1.S1_at) genes are strongly enriched in the uninfected cells. This supports a major physiological role for the uninfected cells in the export of fixed nitrogen in the form of asparagine in Medicago. Interestingly, the uninfected cell “specific” MtbHLH1 transcription factor (Mtr.10993.1.S1_at) was shown to be required for expression of the uninfected cell enriched asparagine synthase (Mtr.8499.1.S1_at), suggesting that it might bind to its promoter [47]. Furthermore, several putative amino-acid transporters, potentially involved in the transport of amino-acids to or from the infected cells, appear to be enriched in the uninfected cells of the nodule (Table S11).

Analysis of the “uninfected cell enriched” gene set indicated a relatively high number of genes, compared to the infected nodule cells, that can be associated with biotic stress or defense responses against pathogenic microbes according to Mapman classification [87] (Figure S1a,b). This supports the hypothesis that suppression of defense responses in the infected cells is essential to allow the accommodation of the rhizobia. Among these genes are several key enzymes involved in phenylpropanoid metabolism (Figure S1e,f) and in jasmonic acid biosynthesis (Figure S1c,d), including several lipoxygenase (*LOX*) genes (Mtr.30415.1.S1_s_at, Mtr.37265.1.S1_at, Mtr.46864.1.S1_at, Mtr.8462.1.S1_at). LOX gene expression correlates with jasmonate levels and LOX protein and transcripts have been detected in the uninfected cells of *Phaseolus vulgaris* and pea nodules [88], [89]. Jasmonates have emerged as important signals in both beneficial and pathogenic plant-microbe interactions and show a complex interplay with Nod factor signaling and the plant hormones salicylic acid and ethylene [90]–[93]. Therefore, jasmonates may play a key role in the development of uninfected cells by controlling defense responses or by affecting the formation of secondary signals required for symbiosis through their effect on secondary metabolism or signaling [93].

### Transcriptional Regulators in Nodule Cell Types

To identify potential key transcriptional regulators in the different cell types we looked for cell-type enriched/specific transcription factors. These are summarized in Table S12.

73 genes appear to be specifically enriched in the meristem of the nodule, representing various TF families. As mentioned earlier TFs related to auxin signaling (*AUX/IAA* and *ARFs*) are enriched in the nodule meristem, as well as various homeobox domain containing TFs, which may be key regulators of nodule meristem organization.

Among the 7 TFs enriched in the distal infection zone are the above described AP2/ERF transcription factors *ERN1* and *ERN2* as well as an additional uncharacterized AP2/ERF TF (Mtr.17511.1.s1_at). Also a gene (Mtr.1584.1.S1_at) encoding a CCAAT-domain binding transcription factor of the HAP2 type appears to be specifically expressed in the distal infection zone, similar to the MtHAP2-1 *TF* controlling symbiosome formation [93]. *In situ* hybridization by Combier and colleagues [94] suggested that *MtHAP2-1* (Mtr.43750.1.s1_at) is most strongly expressed in the meristem of the nodule, however in the LCM array data it appears to be most enriched in the distal infection zone. This might be due to the control of *MtHAP2-1* expression by miRNAs that could leave transcript that can be detected via GeneChip hybridizations.

Most striking among the 5 proximal infection zone enriched TFs are two A-type cytokinin response factors (Mtr.9656.1.s1_at, Mtr.17273.1.s1_s_at), including *MtRR4* which was shown to be regulated by the transcription factor EFD [36]. Both genes are already induced in the distal infection zone, but show the strongest enrichment in the proximal infection zone. As discussed above, this suggests that down-regulation of cytokinin signaling in the (proximal) infection zone is important to allow differentiation and the proper development of the symbiosomes.

Among the 54 putative TFs that are specifically enriched in the infected cells of the fixation zone, there may be key regulators of the infected cell-specific transporter genes (Table S9) and of the characteristic metabolism that facilitates rhizobial nitrogen-fixation. Strikingly, almost 3 times more TFs (143) appear to be specifically enriched in the uninfected cells of the fixation zone, including notably 16 AP2/ERF TFs, 14 Homeobox domain TFs and 7 *SCARECROW*-like GRAS-type TFs. This relatively high number of uninfected cell enriched transcriptional regulators highlights the important role of this cell-type in nodule functioning.

### Conclusion

Here we present a comprehensive gene expression map of an indeterminate Medicago nodule, covering the nodule meristem, (distal and proximal) infection zone as well as infected and uninfected cells from the fixation zone. Our LCM array data fit very well with published gene expression profiles and several cell/tissue specific genes were experimentally verified, indicating that the data may be used as digital “*in situ*”. Many nodule-specifc processes that are essential for a successful nitrogen fixing symbiosis, such as symbiosome formation, differentiation and maintenance, nodule meristem development, nodule cell differentiation (infected *vs* uninfected cells), and metabolite transport processes in the nodule are still far from understood. Therefore, the cell- and tissue-specific data sets presented here offer a valuable resource for further functional studies.

## Materials and Methods

### Plant Growth and Infection

*Medicago truncatula* accession Jemalong A17 was used. Nodulation was done according to Limpens *et al.,* 2004 using a 2 ml suspension (OD_600_ 0.1) of *Sinorhizobium meliloti* strain Sm2011 per plant in (agra)perlite saturated with nitrate-free Fähraeus medium. Three weeks after inoculation nodules were harvested for LCM. *Agrobacterium rhizogenes* mediated root transformation were performed as described by Limpens *et al.* [95], using A. *rhizogenes* strain MSU440.

### Laser Capture Microdissection

Three week-old nodules were fixed in Farmer’s fixative (3∶1 ethanol:acetic acid), after 30 min. vacuum, at 4°C overnight. Fixed nodules were further dehydrated through an ethanol series: 75%, 85%, 100% (4x) for 15 min. each at room temperature (RT). At the first 100% ethanol step eosin B was added to facilitate the recognition of the nodule meristem during the sectioning steps. Nodules were subsequently infiltrated with xylene: ethanol 1∶3, 1∶1, 3∶1 and finally 100% xylene (3x); 30 min at RT each. Next, the nodules were infiltrated with liquid filtered paraffin (Paraplast) at 60°C for 2 days including 4 changes of paraffin. After solidification, 8 µm sections were cut on a RJ2035 microtome (Leica Microsystems, Rijswijk, The Netherlands). Only those consecutive sections that contained a well-developed nodule meristem (based on eosin B staining observed using a binocular) were subsequently deparaffinized using 100% xylene 2×5 min. each, air dried and immediately used for laser capture using a PixCell II LCM system (Arcturus). For each biological replicate, 8 consecutive sections containing ∼50 cells/section were collected and used for RNA isolation. Sections that showed a distorted nodule ontology were discarded. Three biological replicates were collected per cell/tissue-type.

### RNA Extraction and GeneChip Hybridizations

The Qiagen RNeasy Micro kit was used for RNA isolation according to manufacturer’s instructions, with one modification: for the LCM captured cells 50 ng poly-Inosine was added to 350 µl RLT buffer as carrier RNA. On-column DNAse treatment was performed according to the manufacturer’s recommendation. The amount and quality of the RNA in the paraffin embedded nodules was verified before laser capture using agarose gel electrophoresis and using a ND-1000 spectrophotometer (NanoDrop Technologies). The amount and quality of the RNA isolated from the LCM samples was too low to be accurately determined using an Agilent 2100 Bioanalyzer due to the added poly-I.

RNA was processed for use on Affymetrix (Santa Clara, CA, USA) Medicago GeneChips. Samples were amplified according to the first amplification cycle of the Affymetrix Two-cycle Target Labeling kit user manual. Briefly, total RNA containing spiked-in poly-A+ RNA controls was used in a reverse transcription reaction (Two-cycle Target Labeling kit; Affymetrix, Santa Clara, CA, USA) to generate first-strand cDNA. After second-strand synthesis, double-stranded cDNA was used in a 16 h *in vitro* transcription (IVT) reaction to generate aRNA (Two-cycle Target Labeling kit). The generated aRNA samples were than processed according to the Affymetrix GeneChip 3′ IVT Express kit user manual. Briefly, 100 ng of aRNA was used in a reverse transcription reaction (GeneChip 3′ IVT Express Kit; Affymetrix, Santa Clara, CA, USA) to generate first-strand cDNA. Double-stranded cDNA obtained by second-strand synthesis was then used in a 16 h IVT reaction to generate aRNA (GeneChip 3′ IVT Express Kit). Size distribution of *in vitro* transcribed aRNA and fragmented aRNA, respectively, was assessed via an Agilent 2100 Bioanalyzer (Agilent, Böblingen, Germany), using an RNA 6000 Nano Assay. 30 µg to 40 µg of fragmented aRNA was added to a 250-µl (final volume) hybridization cocktail containing hybridization controls. 200 µl of the mixture was hybridized on GeneChips for 16 h at 45°C. Standard post-hybridization wash and double-stain protocols (FS450_0001; GeneChip HWS kit; Affymetrix, Santa Clara, CA, USA) were used on an Affymetrix GeneChip Fluidics Station 450. GeneChips were scanned on an Affymetrix GeneChip scanner 3000 7G.

Packages from the Bioconductor project [96] were used to analyze the array data according to Liu *et al.* [97]. Only the 5 most 3′located probe sets on the GeneChip were used to account for observed 3′bias. To identify genes enriched in a particular LCM sample, genes were first selected that show enriched expression, at least 2-fold higher (p<0.01, q <0,1 ), compared to the average of all other LCM samples. An intensity-based moderated T-statistic (IBMT) [98] was used to calculate p-values and q-values corrected for multiple testing [99]. The obtained (relative) expression values were further analyzed using Microsoft Office Excel 2007 software. Genes that showed ≥2x enrichment compared to all other samples were selected as “cell-type enriched” genes. Expression data were further compared to expression data obtained from the Medicago Gene Expression Atlas (http://mtgea.noble.org/v2/; [22]) and data (24 h NF treatment) published by Czaja et al. [25]. Venn diagrams were created using Venny software (http://bioinfogp.cnb.csic.es/tools/venny/index.html). MapMan software (version 3.5.1) (http://mapman.gabipd.org/web/guest/mapman) was used to analyze gene profiles using the Mt_AFFY_Mt3.1_0510 mapping.

The complete dataset is available from the Gene Expression Omnibus, under accession GSE43354.

### Promoter-GUS Analyses

Putative promoter regions were PCR amplified from Medicago genomic DNA using Phusion high fidelity Taq polymerase (New England Biolabs) and directionally cloned into pENTR-D-TOPO (Invitrogen). The following primers were used: MtLYK3p-F CACCTGAATCAAGAAGAGAGAGAGAAAGAG, MtLYK3p-R AGCCAAGTACATGAGATTGGATAA; MtROP2p-F CACCTAGCTTTATCACACACAAATGTCCC, MtROP2p –R ATTGTATAAATGGAACTAAGGTTTTGTTG; MtENOD8.2p-F CACCTCAATAGGGCATGTTACAAAAAGTG, MtENOD8.2p-R GAATTTCATGAAGCACAAAGGAAC.

After sequence analysis, the corresponding pENTR clones were used to recombine the promoters into pKGWFS2-RR [100], creating a promoter:GUS-GFP reporter fusion. Transgenic roots were obtained via *A. rhizogenes* mediated root transformation. Histochemical GUS staining was performed according to [100] up to 4 hours in 0.1 M phosphate buffer pH7.0 containing, 3% sucrose, 5 µM EDTA, 0.5 mM K4Fe(CN)6, 0.5 mM K3Fe(CN)6 and 1 mM X-gluc (dissolved in DMFO) at 37°C. Subsequently, the GUS stained nodules were fixed using 5% glutaraldehyde (in 0.1 M phosphate buffer) and embedded in technovit 7100 (Heraeus-Kulzer) according to the manufacturers protocol. 10 µm sections were cut using a Reichert-Jung 2035 microtome, counterstained with 0.1% ruthenium red, and analyzed using a Leica DM5500B microscope equipped with a Leica DFC425C camera (Leica microsystems, Germany).

### *In situ* Hybridization

*In situ* hybridization of Medicago nodules were performed as described by Limpens et al. [43], based on the protocol by van de Wiel *et al.* [101]. A 220 bp ENOD12 cDNA fragment was amplified using primers: ENOD12-iF (AGGCATCCTCCAGCAGAAGA), ENOD12-iR (ATAGCACGATTTTACACTCATACCTCATA), and cloned into the pCRII-Blunt TOPO vector (Invitrogen). T7 and Sp6 primers were used to synthesize radioactively label (sense and antisense) RNA probes after digesting the plasmids with NotI (sense) or SpeI (antisense). For hybridization 2×10^6^ cpm 35S (sense and antisense) labeled probe was used.

## Supporting Information

Figure S1 Schematic representation of genes specifically enriched (filled squares; selected ≥2 enriched) in infected (a,c,e) and uninfected (b,d,f) cells from the fixation zone according to Mapman v.3.5.1 classification (Mt_AFFY_Mt3.1_0510 mapping). (a,b) Genes potentially associated with biotic stress. (c,d) Genes associated with jasmonic acid synthesis. (e,f) Genes involved in phenylpropanoid metabolism.
(TIF)

Table S1 Gene expression “enrichment” in a particular LCM sample compared to the average of all other samples, based on the five most 3′ located Medicago truncatula probe sets on the Medicago GeneChips. Column A: Medicago GeneChip Id; Column B: gene name; Column C: Gene Annotation; Column D: Mean expression value (log2) in all LCM samples; Column E–V: fold enrichment for each LCM cell/tissue including p and q (corrected for multiple testing) statistics and average signal intensity (A) from three biological replicates; Column E–G = Meristem, H–J = distal infection zone. K–M = Proximal infection zone, N–P = distal+proximal infection zone, Q–S = Infected cells from the fixation zone, T–V = Uninfected cells from the fixation zone. Column W–Z: log2 expression values from the Medicago Gene Expression Atlas (http://mtgea.noble.org/v2/), representing Column W = root, X = 4 day old nodules, Y = 10 day old nodules, Z = 14 day-old nodules. Column AA: Gene Ontology (GO) Biological function ID, Column AB: Biological function GO term, Column AC: Molecular Function GO ID, Column AD: Molecular function GO term, Column AE: prediced gene function based on EuKaryotic Orthologous Groups.
(XLSB)

Table S2 Genes showing enriched expression specifically in the nodule meristem.
(XLS)

Table S3 Genes showing enriched expression in the infection zone of the nodule.
(XLS)

Table S4 Genes showing enriched expression specifically in the distal infection zone.
(XLS)

Table S5 Genes showing enriched expression specifically in the proximal infection zone.
(XLS)

Table S6 Genes showing enriched expression specifically in the infected cells of the fixation.
(XLS)

Table S7 Genes showing enriched expression specifically in the uninfected cells of the fixation zone.
(XLS)

Table S8 Selected genes showing 2 fold upregulation upon 24 h Nod factor treatment, based on Czaja et al., 2012 [25].
(XLS)

Table S9 Putative (receptor) protein kinase genes showing enriched expression specifically in the nodule meristem (sheet 1), distal infection zone (sheet 2), proximal infection zone (sheet 3), total infection zone (sheet 4), infected cells from the fixation zone (sheet 5) and uninfected cells (sheet 6).
(XLS)

Table S10 Genes encoding putative transporters enriched in the infected cells of the fixation zone.
(XLS)

Table S11 Genes encoding putative transporters enriched in the uninfected cells of the fixation zone.
(XLS)

Table S12 Transcription factor genes showing enriched expression specifically in the meristem (sheet 1), distal infection zone (sheet 2), proximal infection zone (sheet 3), total infection zone (sheet 4), infected cells from the fixation zone (sheet 5) and uninfected cells (sheet 6).
(XLS)

Table S13 Genes showing enriched expression in mycorrhized roots, according to the Medicago Gene Expression Atlas (http://mtgea.noble.org/v2/).
(XLS)
