# Correction: Cell- and Tissue-Specific Transcriptome Analyses of *Medicago truncatula* Root Nodules

**DOI:** 10.1371/annotation/d8bce646-4127-410a-b4dd-d2d2779a4745

**Published:** 2014-01-06

**Authors:** Erik Limpens, Sjef Moling, Guido Hooiveld, Patrícia A. Pereira, Ton Bisseling, Jörg D. Becker, Helge Küster

In the Results and Discussion section under 'Cell-type Specific Expression Profiling of Medicago Root Nodules' the following sentence was erroneously included: "Figure 2 summarizes the number of genes that show at least 2 fold enriched expression compared to the average of the other LCM samples."

Under 'Validation of Cell-type Specific Expression in Medicago Root Nodules', the last sentence of the first paragraph incorrectly refers to Table 2. The corrected sentence is: "Additionally, we analyzed the expression profile of several selected genes in the nodule (Figure 2)."

