# Supplementary figures and images for: Cell- and Tissue-Specific Transcriptome Analyses of Medicago truncatula Root Nodules
Source: PLoS One. 2013 May 29;8(5):e64377. doi: 10.1371/journal.pone.0064377 (PMC3667139; doi:10.1371/journal.pone.0064377)

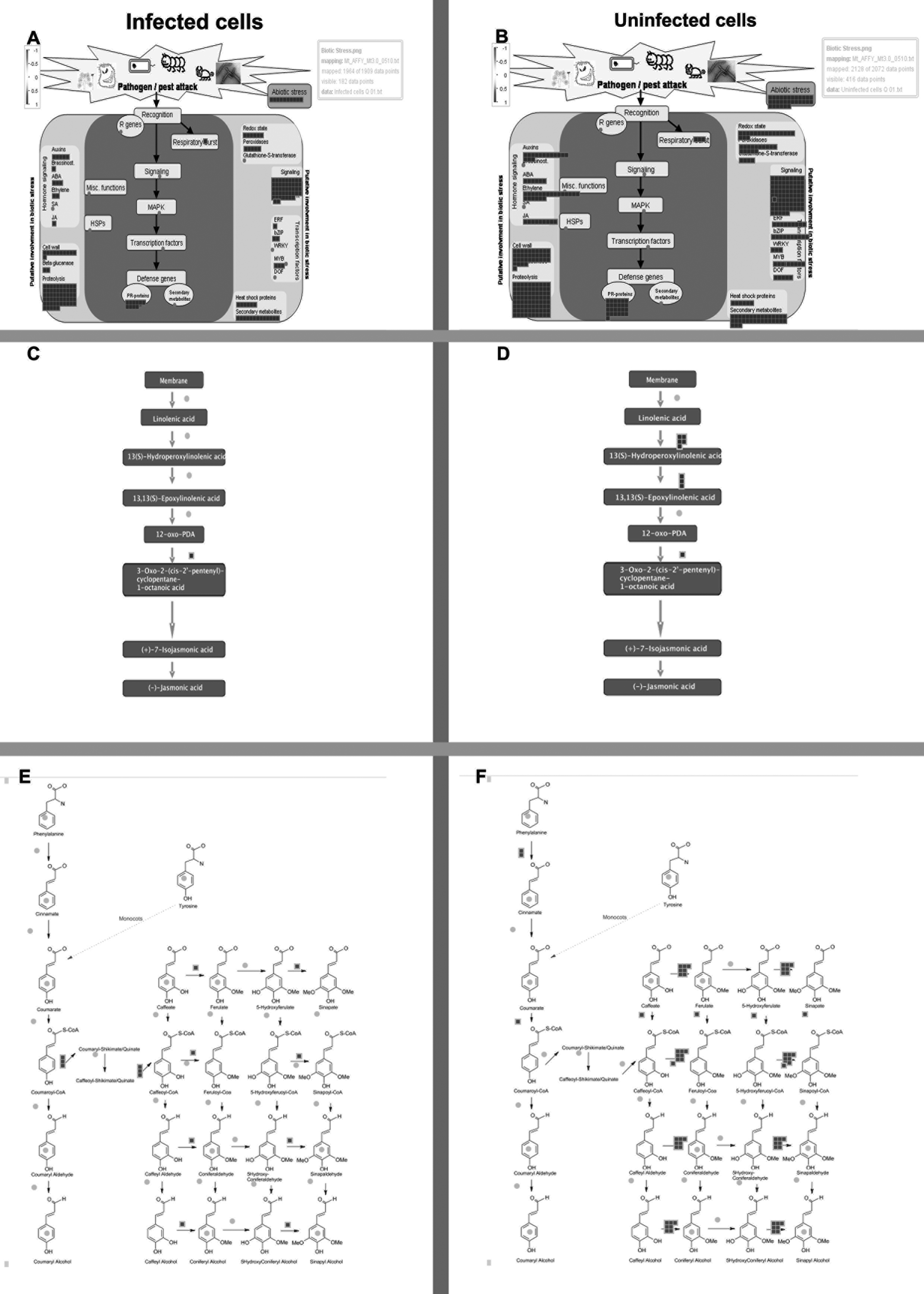

Supplement: Figure S1 — Schematic representation of genes specifically enriched (filled squares; selected ≥2 enriched) in infected (a,c,e) and uninfected (b,d,f) cells from the fixation zone according to Mapman v.3.5.1 classification (Mt_AFFY_Mt3.1_0510 mapping). (a,b) Genes potentially associated with biotic stress. (c,d) Genes associated with jasmonic acid synthesis. (e,f) Genes involved in phenylpropanoid metabolism. (TIF) [file pone.0064377.s001.tif]
